# Catamenial Pneumothorax as an Underrecognized Manifestation of Thoracic Endometriosis: A 25-Year Single-Center Experience

**DOI:** 10.3390/jcm15134941

**Published:** 2026-06-25

**Authors:** Henrike Deissner, Benedikt Niedermaier, Raffaella Griffo, Cosmas Wimmer, Markus Polke, Franziska C. Trudzinski, Florian Eichhorn, Marc A. Schneider, Kadriya Yuskaeva, Hauke Winter, Laura V. Klotz

**Affiliations:** 1 Department of Thoracic Surgery, Thoraxklinik, Heidelberg University Hospital, 69126 Heidelberg, Germany; 2 Translational Lung Research Center Heidelberg (TLRC), German Center for Lung Research (DZL), 69120 Heidelberg, Germany; 3 Center for Interstitial and Rare Lung Diseases, Pneumology and Critical Care Medicine, Thoraxklinik, Heidelberg University Hospital, 69126 Heidelberg, Germany; 4 Translational Research Unit (STF) Thoraxklinik, Heidelberg University Hospital, 69126 Heidelberg, Germany

**Keywords:** thoracic surgery, spontaneous pneumothorax, gender, recurrence, talc pleurodesis, diaphragm, thoracic endometriosis, catamenial pneumothorax

## Abstract

**Objectives**: Catamenial pneumothorax (CP) is a rare but clinically relevant cause of spontaneous pneumothorax (SP) in women and is associated with high recurrence rates. We hypothesized that CP is underrecognized in routine surgical practice due to an incomplete clinical assessment rather than an absence of characteristic intraoperative findings. **Methods**: We conducted a retrospective single-center analysis of all patients undergoing surgical treatment for pneumothorax between 2000 and 2025. Female patients with SP and no structural lung disease were identified and systematically evaluated for features suggestive of CP. Demographic, clinical, intraoperative, and outcome data were compared between patients with and without CP. **Results**: Among 4581 surgically treated pneumothoraces, 1253 (27.4%) occurred in women. Of these, 211 cases of SP without structural lung disease were analyzed. CP was identified in 15 cases among 14 patients (7.1%). Patients with CP were older at initial diagnosis (median 39 vs. 32 years; *p* = 0.0264) and exhibited higher recurrence rates (92.9% vs. 42.4%; *p* = 0.0003). A temporal association with menstruation was documented in 57.1% of CP cases, while in 35.7% no such assessment had been performed. Intraoperative findings suggestive of thoracic endometriosis were present in 85.7% of CP patients, whereas histological confirmation was achieved in only 14.3%. **Conclusions**: CP is likely underdiagnosed in surgical cohorts of women with SP. The principal diagnostic limitation appears to be incomplete history-taking rather than lack of intraoperative evidence. Given the high recurrence risk and limited efficacy of surgery alone, systematic assessment of menstrual association and interdisciplinary management are essential to optimize outcomes.

## 1. Introduction

Primary spontaneous pneumothorax is defined as a non-traumatic collapse of the lung without underlying structural lung disease and is associated with a considerable risk of recurrence and potential morbidity [1]. The incidence of spontaneous pneumothorax (SP) is significantly higher in men than in women, with reported rates of 24.0 per 100,000 in men and 9.8 per 100,000 in women [2,3]. Notably, age distribution differs between sexes, with an earlier peak in men and a later peak in women [1,4,5]. The overall recurrence rate averages 20–30%, although rates as high as 83% have been reported in individual studies [1,2]. Thoracoscopic wedge resection combined with pleurodesis can markedly reduce the risk of recurrence to approximately 5% [1,6].

Catamenial pneumothorax (CP) is a specific subtype of SP in women of reproductive age, defined by its temporal association with menstruation. CP is commonly attributed to thoracic endometriosis and typically presents on the right side [7,8,9]. Several mechanisms have been proposed to explain thoracic endometriosis. Endometrial cells may reach the right hemithorax through diaphragmatic fenestrations and migrate into the thoracic cavity, possibly facilitated by preferential peritoneal fluid flow toward the right upper abdomen [9,10,11]. Additional hypotheses include lymphatic or hematogenous dissemination and hormonally mediated cyclical changes in pleural or diaphragmatic tissue [11,12]. Hormonal influences also play a role in promoting cyclical pleural changes and tissue fragility, which can result in pneumothorax during menstruation [13,14]. These interacting mechanisms likely explain the varied clinical and intraoperative manifestations of thoracic endometriosis.

Despite characteristic findings during surgery, such as pleural lesions and diaphragmatic defects, diagnosis remains challenging. The clinical presentation is often nonspecific, and hormonal therapy can mask the cyclical pattern [7,8,9,14]. Pelvic endometriosis frequently coexists with CP [7]. Importantly, CP is associated with a high recurrence rate, even after surgical treatment. Therefore, it requires interdisciplinary management, including a gynecological evaluation and hormonal therapy [9,13,14,15]. However, real-world data on the frequency of missed CP diagnoses in surgical cohorts remains scarce.

The aim of this study was to investigate SP in women without structural lung disease, with a particular focus on identifying features suggestive of CP. We hypothesized that CP is underrecognized in routine surgical practice due to an incomplete clinical assessment rather than an absence of characteristic findings.

## 2. Materials and Methods

This retrospective, single-center study included all patients who underwent surgical treatment for pneumothorax at our high-volume thoracic surgery center between January 2000 and August 2025. Patients were stratified by sex. Female patients with SP without evidence of structural lung disease and aged ≤ 50 years were identified and evaluated further for features suggestive of CP. The study was approved by the institutional ethics committee, waiving the requirement for individual informed consent (S-174/2019).

Standard management included chest tube insertion for symptomatic pneumothorax, typically in the Bülau position. For cases with mild symptoms, conservative treatment or primary thoracoscopic intervention was considered. To prevent surgical recurrence, thoracoscopic pleurodesis was performed, primarily via pleurectomy. Talc pleurodesis was applied in cases of recurrence. Intraoperatively, systematic inspection of the diaphragm, pleura, and lung parenchyma was performed. Suspected lesions were biopsied where feasible. Particular attention was paid to diaphragmatic defects and pleural endometriotic lesions. Preoperative thoracic CT scans were routinely performed to assess structural abnormalities. Catamenial pneumothorax was defined based on a combination of clinical and intraoperative criteria, including a temporal association with menstruation (within ±72 h), the presence of characteristic intraoperative findings, or both.

Data collected included demographic characteristics, clinical presentation, intraoperative findings, and treatment outcomes. Continuous variables are presented as medians with interquartile ranges (IQR), while categorical variables are presented as counts and percentages. Group comparisons were performed using the Mann–Whitney U test or chi-square test, as appropriate. A *p*-value < 0.05 was considered statistically significant. All statistical procedures were performed using GraphPad Prism version 10.6.1 and Microsoft Excel.

## 3. Results

**Figure 1:**
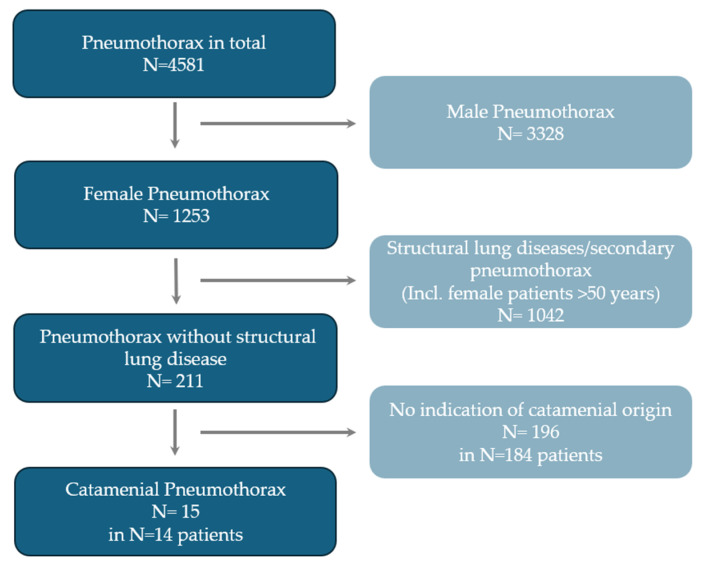
**Flow diagram of patient selection**. Among 4581 surgically treated pneumothoraces, 211 cases occurred in women without structural lung disease. Catamenial pneumothorax was identified in 15 events among 14 patients.

Over a 25-year period, a total of 4581 cases of pneumothorax requiring surgical intervention were identified at our high-volume center, of which 3328 (72.6%) occurred in men and 1253 (27.4%) occurred in women. Among the female patients, 83.2% had secondary pneumothorax associated with structural lung disease. The remaining 211 cases of SP without structural lung disease were analyzed for CP (Figure 1). Fifteen pneumothorax events in 14 patients (7.1%) met the diagnostic criteria for CP. The remaining 196 cases (92.9%) corresponded to 184 patients and showed no evidence of a catamenial etiology. These cases served as the comparison group.

### 3.1. Patients

#### 3.1.1. Control Cohort

Among the 184 women without structural lung disease, the median age at initial diagnosis was 32.0 years (interquartile range (IQR) 23.3–40.0). A total of 56 (30.4%) were current smokers, 17 (9.2%) smoked in the past, and 36 patients were never smokers (19.6%). The median body mass index (BMI) was 20.7 (IQR: 18.8–22.3). Right-sided pneumothorax occurred in 114 (57.6%) and left-sided pneumothorax in 71 (35.9%) women. Bilateral pneumothorax occurred in 13 (6.6%) patients.

#### 3.1.2. Catamenial Pneumothorax Cohort

CP was identified in 15 cases among 14 patients. Thirteen of these cases were right-sided. One patient presented with a left-sided CP, which required the insertion of a chest tube. The median age of the patients at initial diagnosis was 39 years (interquartile range (IQR): 32.5–40.8). The median BMI was 21.6 (IQR: 21.1–22.2) and the median Eastern Cooperative Oncology Group (ECOG) performance status was 0. A positive smoking history was present in four patients (28.6%). Patients with CP were significantly older at initial diagnosis (median 39 vs. 32 years in non-CP patients; *p* = 0.0264). Right-sided pneumothorax predominated in patients with CP (85.7% vs. 55.4%; *p* = 0.0625). Patient characteristics are detailed in Table 1.

Four patients (28.6%) were diagnosed with endometriosis elsewhere. A temporal association with menstruation was documented in 57.1% of CP patients, while no such assessment had been performed in 35.7%. One patient (7.1%) reported no observable association, though she was using a hormonal intrauterine device. Seven (50.0%) patients were using hormonal contraceptives at the time of the initial event. Intraoperative findings suggestive of thoracic endometriosis were present in 85.7% of CP patients, whereas histological confirmation was achieved in only 14.3% of patients.

**Figure 2:**
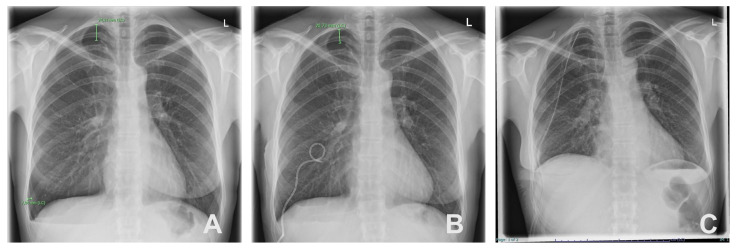
**X-ray images of a female patient who suffered spontaneous pneumothorax for the first time at the age of 40.** (**A**) Third recurrence at the age of 48. (**B**) Initial treatment using CT-guided drainage following pleurectomy. (**C**) Fully expanded lung following Re-VATS with talc pleurodesis.

**Figure 3:**
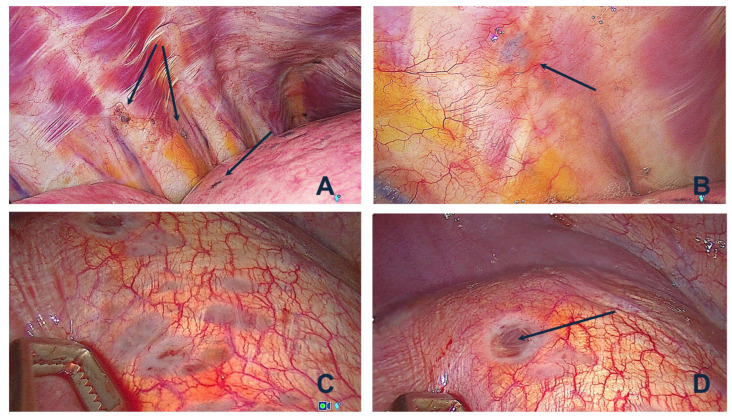
**Intraoperative findings of a patient with third recurrence of SP at the age of 48 years.** (**A**,**B**): Pleural endometriotic lesions; (**C**): Diaphragm gaps; (**D**): Free-floating liver under a diaphragmatic gap.

Patients with CP had a higher number of pneumothorax episodes, with a median of three. Talc pleurodesis was performed on 11 patients (78.6%) in cases of failed pleurectomy. Two of these patients underwent repeated talc pleurodesis. Five patients (35.7%) had targeted CT-guided drainage insertion due to symptoms caused by adhesions after pleurectomy and talc pleurodesis (Figure 2). Perioperative characteristics are shown in Table 2 and Figure 3.

Median follow-up time was 16.34 months (IQR: 5.8–66.0 months). Median recurrence-free survival was 13.28 months (IQR: 4.7–66.0 months) for patients with CP. Multivariate Cox regression analysis showed that a higher BMI was associated with a reduced risk of recurrence and had borderline significance (HR 0.706; 95% CI 0.495–1.005; *p* = 0.053). Age, ECOG status and smoking status had no significant independent influence on recurrence-free survival for patients with CP. However, the statistics are limited due to the small number of patients, which makes it difficult to draw meaningful conclusions.

## 4. Discussion

This study demonstrates that CP is likely underrecognized in women undergoing surgical treatment for SP without structural lung disease. Although CP is generally considered rare, our findings suggest that its true prevalence may be underestimated due to diagnostic limitations in routine clinical practice rather than a true absence of the disease [8,11,12]. With a mean age of 39 years at first pneumothorax event, women with CP are significantly older at initial diagnosis. This may be one reason for the higher age peak among women compared to men [14].

A central finding is the substantial gap in clinical history-taking. In more than one-third of patients ultimately classified as having CP, no assessment of a temporal association with menstruation had been performed. In total, 57.1% of patients with CP reported occurrence with menstruation. One did not, but she had a hormonal coil, which can disrupt the natural menstrual cycle and reduce menstrual bleeding. Taking a medical history can be difficult when hormonal contraceptives are used because they may not reflect the typical presentation of catamenial pneumothorax [16]. Even in the presence of characteristic intraoperative findings, failure to obtain a targeted history may prevent accurate classification [5,11,17]. Our data therefore indicate that underdiagnosis of CP is primarily driven by insufficient clinical assessment rather than a lack of suggestive findings. In contrast, intraoperative exploration often revealed abnormalities indicative of thoracic endometriosis, such as pleural lesions and diaphragmatic defects [18]. These findings were present in most CP patients and align with previous reports describing right-sided predominance and characteristic diaphragmatic involvement [19]. Our data indicate that pathognomonic intraoperative findings, such as pleural endometriotic lesions (78.6%) and diaphragmatic defects (64.3%), are commonly observed in patients suspected of having CP. However, despite clear intraoperative changes, histopathological evidence of endometriotic lesions was rarely found (14.3%). This further complicates the already difficult task of making an accurate diagnosis. Several pathophysiological mechanisms have been proposed to explain these observations, including transdiaphragmatic passage of endometrial tissue, microembolization, and hormonally mediated changes in pleural integrity [9,10,13,14]. The predominance of right-sided disease is commonly attributed to the heart and pericardium’s protective effect on the left hemidiaphragm and clockwise peritoneal fluid circulation [7,9,10]. Our findings are consistent with these concepts, as most CP cases in our cohort occurred on the right side.

Despite the frequent occurrence of intraoperative abnormalities, histopathological confirmation was achieved in only a small proportion of cases. This discrepancy highlights a well-known limitation and suggests that relying on histology alone can result in underdiagnosis of CP [13,14,19]. Therefore, a combined diagnostic approach that integrates clinical history, intraoperative findings, and contextual factors appears essential. Due to the difficulty of making clinical and histopathological diagnoses, it is impossible to rule out a significant number of unrecognized cases [12,14,17]. Importantly, CP was associated with a higher recurrence rate than non-catamenial SP, which emphasizes its clinical relevance. Recurrent pneumothorax increases morbidity and may require repeated surgeries, which are often more technically challenging due to adhesions and prior pleurodesis. These findings underscore the inadequacy of thoracic surgery alone as a definitive treatment strategy for CP and highlight the necessity of interdisciplinary management with gynecology [7,9,13,14,15]. Based on our findings, a structured diagnostic and therapeutic approach appears warranted. For women of reproductive age presenting with SP and no evidence of structural lung disease, a systematic assessment of their menstrual history should be performed routinely. If CP is suspected, the intraoperative inspection should explicitly focus on the diaphragm and pleura, paying particular attention to fenestrations and endometriotic lesions [20]. The presence of typical intraoperative findings combined with a suggestive clinical history should prompt consideration of CP, even in the absence of histological confirmation. Early involvement of a gynecologist and initiation of adjunctive hormonal therapy should be considered to reduce the risk of recurrence. To date, the evidence base for the use of hormonal therapy alongside CP is observational, with no prospective data available [10,13]. From a broader perspective, these findings support the importance of incorporating sex-specific considerations into thoracic surgical practice. Although SP is more prevalent in men, the underlying etiologies in women can differ significantly and necessitate customized diagnostic approaches.

This study has several limitations. Its retrospective and single-center design may limit its generalizability. The relatively small number of CP cases reflects the rarity of the condition but also restricts statistical power. Additionally, repeated surgical interventions due to persistent air leakage in the postoperative course were also included and may not necessarily indicate recurrence of pneumothorax. Nevertheless, the large overall cohort provides robust real-world insight into current diagnostic practice.

## 5. Conclusions

In conclusion, CP should be considered more often for women with SP who do not have structural lung disease. Our data suggest that the condition is rare and underdiagnosed, primarily due to incomplete clinical evaluation. Improving patient outcomes and reducing recurrence rates may be possible by addressing this gap through structured history-taking, targeted intraoperative assessment, and interdisciplinary management.

## Figures and Tables

**Table 1:** Comparison of female patients with spontaneous pneumothorax without structural lung disease: non-catamenial vs. catamenial pneumothorax. BMI—body mass index, ECOG—Eastern Cooperative Oncology Group status.

Variable	Non-CP (n = 184)	CP (n = 14)	*p*-Value
**Age at first diagnosis, years, median (IQR)**	32.0 (23.3–40.0)	39.0 (31.2–41.2)	0.0264
**Height, m, median (IQR)**	1.68	1.67	0.4557
**Body weight, kg, median (IQR)**	58.0	58.0	0.2658
**BMI, median (IQR)**	20.7 (18.8–22.3)	21.6 (21.1–22.2)	0.0309
**Smoking status, n (%)**			0.0839
current	56 (30.4)	4 (28.6)	
former	17 (9.2)	1 (7.1)	
never	36 (19.6)	5 (35.7)	
unknown	75 (40.8)	4 (28.6)	
**ECOG performance status, n (%)**			0.6302
0–1	181 (98.4)	14 (100)	
>1	3 (1.6)	0 (0)	
**Pneumothorax location, n (%)**			0.0625
right	102 (55.4)	12 (85.7)	
left	70 (38.0)	1 (7.1)	
bilateral	12 (6.5)	1 (7.1)	
**Recurrence, n (%)**	91 (42.4)	13 (92.9)	0.0003

**Table 2:** Perioperative characteristics of patients with catamenial pneumothorax. VATS—video-assisted thoracoscopy, CT—computed tomography.

Variable	Value
**Clinical characteristics**	
extrathoracic endometriosis, n (%)	4 (28.6)
hormonal contraceptive use, n (%)	7 (50.0)
menstrual association, n (%)	
present	8 (57.1)
not assessed	5 (35.7)
absent	1 (7.1)
**Pneumothorax characteristics**	
pneumothorax events, n, median (IQR)	3 (2–4)
total pneumothorax events, n	45
total surgical interventions, n	53
**Intraoperative findings**	
bullae, n (%)	5 (35.7)
diaphragmatic defects, n (%)	9 (64.3)
pleural endometriotic lesions, n (%)	11 (78.6)
histological confirmation, n (%)	2 (14.3)
**Treatment (total interventions = 53)**	
chest tube insertion, n (%)	21 (39.6)
VATS pleurectomy, n (%)	14 (26.4)
VATS talc pleurodesis, n (%)	13 (24.5)
CT-guided drainage, n (%)	5 (9.4)
conservative management, n (%)	2 (3.8)

## Data Availability

Raw data can be provided on reasonable request due to patient privacy regulations.

## Abbreviations

The following abbreviations are used in this manuscript:

BMI	Body Mass Index
CP	Catamenial Pneumothorax
CT	Computed Tomography
EGOG	Eastern Cooperative Oncology Group status
IQR	Interquartile Range
SP	Spontaneous Pneumothorax
VATS	Video-assisted thoracoscopy
